# Early Drop-Out from Sports and Strategic Learning Skills: A Cross-Country Study in Italian and Spanish Students

**DOI:** 10.3390/sports9070096

**Published:** 2021-06-30

**Authors:** Carla Consoni, Caterina Pesce, Domenico Cherubini

**Affiliations:** 1 Sports Faculty, UCAM Catholic University of Murcia, 30107 Murcia, Spain; dcherubini@ucam.edu; 2 Department of Movement, Human and Health Sciences, University of Rome “Foro Italico”, 00135 Rome, Italy; caterina.pesce@uniroma4.it

**Keywords:** physical activity, strategic skills, affective, motivational, sport habits

## Abstract

The search for overarching factors involved in both sport and broader lifestyle and achievement domains may help to understand the early drop-out phenomenon. This study aimed to analyze the association between early sport drop-out and strategic learning skills, checking for the individual and joint role of nationality, school type, gender, age and sport habits. Six hundred and fourteen Italian and Spanish students aged 14–18 years completed two self-assessment questionnaires concerning physical activity, sports habits and learning strategies. Outcomes were analyzed with frequency analysis. Higher affective–motivational strategic learning skills were associated with lower drop-out rates in Italian but not Spanish students. In high schools with an enhanced sports curriculum, drop-out rates were negligible compared to other Italian and Spanish curricula. A lack of persistence in the same sport type was significantly associated with a higher drop-out rate in males but not in female students, who had overall higher drop-out rates. This study suggests that overarching personal skills, cultural characteristics and sports habits may independently and jointly contribute to sport drop-out. Specifically, affective–motivational learning skills may play a key role in sport persistence and in strategies tailored to drop-out prevention.

## 1. Introduction

Physical and sports activities provide benefits to physical, mental and socio-emotional health [1]. All people who exercise regularly improve their quality of life at any age and without gender differences [2,3]. Moreover, in young people, sports training promotes good health, an optimal psycho-physical development and the acquisition of healthy lifestyles that will persist even in later ages [4]. Generally, children start playing sports to socialize, improve motor skills and have fun [5]. The more the approach is enjoyable and gratifying, the stronger the motivation to continue doing sports over time and the probability to ensure greater investment in sport and talent identification [6,7].

Nevertheless, young athletes, especially the talented ones, may quit the sport prematurely during their school education before reaching their potential peak performance due to personal, social and contextual factors [8,9]. The combination of socioeconomic [10] and parental/coaches/peers support factors can predict sports participation/abandonment during childhood [11]. Therefore, sports drop-out has been identified as a multifactorial and complex phenomenon, strongly influenced by different cultural backgrounds [10] and behavioral factors, as well as by personal characteristics, types of sports, attitudes and motivations [12]. The drop-out rates from sports gradually increase across adolescence [13]; this is associated with physical inactivity later in life, thus contributing to unhealthy lifestyles [14]. Conversely, young people who exercise keep healthy lifestyle habits, such as continued physical activity and healthy nutrition [15]. Findings in Sport and Physical Activity surveys [16,17] showed that the percentage of young people aged 15–24 years who exercise regularly decreased in ten years from 14% to 9%. In this group, 15% were males and 33% females (expressed in percentage of the whole sample interviewed: 15–55 years). In addition, several authors confirmed the drop-out rates from sports being higher in females than in males [18,19].

Reasons for dropping-out of sports have been classified into performance and training factors, education and work obligations [20,21], motivational factors, social environment and other interests [22,23]. Most recently, Crane & Temple [24] and Witt & Dangi [25] discussed the *leisure constraints theory* [26] and categorized the variables associated with drop-out from sports as intrapersonal, interpersonal and structural constraints. The findings related to this theoretical model showed drop-out rates being associated with sport-specific attrition factors at intra-personal and interpersonal levels. According to the cognitive–affective model [27], as well as Sorkkila et al. [28], demands, critical situations and personal resource deficit are associated with the development of sport and school burnout. On the other hand, Cosh and Tully [29] observed that playing sport could upskill young athletes by enhancing time management, self-care, self-efficacy and specific strategies for coping with stress in different life areas in young people. Moreover, transferring these skills to academic commitment may help young people successfully achieve their goals in other life domains. Adolescent sports participation [30] and, more broadly, physical activity are linked to academic achievement [31,32]. This is consistent with the evidence that systematic exercise programs may actually enhance the development of cognitive and metacognitive skills known to be important in addressing challenges of achievements both academically and beyond [33].

In summary, these studies confirm an important relationship linking sport and physical activity to functional personal resources. Furthermore, these resources have a cross-boundary influence on every learning context and are relevant to dealing with challenges, changes and critical situations. The linkage of cross-functional personal skills in more domains encourages the investigation of unspecific dimensions and factors, which could play a role in drop-out from, or persistence in, sports activity but are still underexplored.

In the framework of the soft skills that have to be promoted in the educational field, Bay, Grządziel and Pellerey [34] define specific learning skills as strategic. These skills are cognitive, metacognitive, affective, motivational and volitional in nature, and refer to the capacity for self-determination and self-regulation. Self-determination is related to motivation, choice and intention of the action in different salient and emotionally laden life conditions. Self-regulation refers to the monitoring, evaluation and control system of an action to monitor its consistency, stability and orientation, and to regulate its functionality [35]. These capacities enable positive behaviour towards challenges and changes; they are functional in order to carry out personal objectives. The strategic learning skills are general and transferable, and seem to constitute stable internal dispositions indispensable to dealing with study, work, sport and any relevant tasks in life autonomously and successfully. From an early age, good management of these skills allows the person to play a self-orienting role in different learning and life domains [36]. In other life contexts, the sport experiences may provide a favorable environment to promote positive youth development through sport participation [37].

While the search for factors associated with sport drop-out rates is ongoing, the role played by skills that may affect performance in broader life domains, different from sport, is still under-investigated. Thus, the main aim of this study was to explore the association between early drop-out from sport in adolescence and strategic learning skills. We hypothesized that these skills functional to learning processes, mainly studied in academic achievement research [38], may also be associated with sport participation and, vice versa, an insufficient level/lack of such skills could be associated to early drop-out from sport. The hypothesis is grounded on indirect evidence of piecemeal associations between strategic learning skills, sport participation and academic achievement [39]. These associations have been mainly studied in a unidirectional manner, focusing on the beneficial effect of the sports activity on other life domains and academic achievement [40,41]. However, it is possible that these relations are bidirectional and the co-variation of sport participation and academic achievement is due to commonalities in the underlying domain-general of functional skills that rely on high-level cognitive function [42,43]. Thus, we expected that strategic learning skills considered functional to academic achievement might also be supportive of sport participation adherence. We tested this hypothesis in an exploratory manner, looking for associations with a cross-sectional design. Since early drop-out seems influenced by demographic variables such as gender and age [23], and by the quantitative and qualitative characteristics of sports activity [44,45], we further explored the hypothesized association of sport drop-out rates with strategic learning skills as a function of these variables. This association was investigated also as a function of nationality in Italian and Spanish adolescents to perform a cross-country comparison and extend the generalizability of results.

## 2. Materials and Methods

### 2.1. Participants

Participants were sampled in a stratified manner, purposefully identifying different types of high schools and urban areas of the cities of Rome (Italy) and Murcia (Spain) before sampling. Both are large cities located in a central region of their respective countries, close to the Mediterranean coast. To reduce possible influences determined by the socio-economic status of students participating in the research, school structures operating in districts characterized by a prevailing middle social status were included in the study. All participants were screened and selected based on three criteria: age (14 to 18 years); high school students; eligible senior high school curriculum. The Spanish participants attended the compulsory secondary school (ESO) that is the unique eligible curriculum until compulsory school. In contrast, Italian students have the option to choose among different school curricula. To the aim of this study, we recruited Italian students who attended upper secondary schools with an enhanced sports curriculum (sports senior high school), and other non-sport enhanced (scientific, applied sciences, linguistic) senior high schools. In this way, we could perform a cross-country comparison between Spanish and Italian students attending non-sport enhanced senior high schools, as well as a comparison of students attending senior high school with an enhanced sport curriculum with the other traditional Italian and Spanish school curricula. Since the Italian students could attend many different high-school curricula, it was deemed appropriate to recruit a larger number of Italian rather than Spanish students to have a representative sample for each type of eligible Italian high school. Thus, the sample consisted of 188 Spanish (109 male, 79 female) and 426 Italian (257 male, 169 female) students aged 14–18 years. The students consented to fill in the questionnaires and their parents/guardians (if younger than 18 years) signed an informed written consent before participating in the study.

### 2.2. Instruments

The students, in their own native language, completed two self-assessment questionnaires after forward–backward translation of the Italian version of the CAPAFD (originally developed in Spanish) and of the Spanish version of the QSA-R (originally developed in Italian).

The first questionnaire asked about the motivation and training of physical-sporting activity: Questionnaire for the Analysis of the Practice of Physical-Sports Activities, CAPAFD [46]. This questionnaire was developed to evaluate physical and sport habits associated with different socio-demographic variables. It has been proven suitable to collect descriptive data on objectively reportable demographics and behavioral habits [46]. The original questionnaire is made of eleven blocks comprising twenty-six questions targeted at people aged 15–64 years. In order to collect information on sport habits and drop-out relevant to the aim of the present study, two sections comprising seventeen questions were selected. These questions included information concerning sport drop-out, sport participation and background variables relevant to the study. Specifically, the selected items allowed collecting information about: age, gender, type of attended school, onset age of sports activity, current/past registration to a sports Federation, sport practice/drop-out, current and past sports practiced (years of practice for each type of sport practiced, if any), workout frequency (hours/days and days/week), competitive sports activity performed (if any) and sport practiced in organized club/association. This information was collected with three types of questions. Five of the 17 items were open-ended questions regarding demographic information (e.g., School attended); 5 items were close-ended questions with dichotomous answer (yes or no; e.g., Have you dropped-out of sports?*)*; the remaining 7 items were multiple choice with three to six answers (e.g., How much time do you spend working out daily on average?*)*.

The second questionnaire, the Questionnaire on Learning Strategies (reduced version) QSA-R validated by Margottini [47], was about students’ strategic competences for learning to self-evaluate their studying habits and/or the critical situations encountered in schoolwork. The QSA-R estimates the perceived mastery of eight strategic skills, grouped in two dimensions of four scales each: cognitive/metacognitive (C1, C2, C3, C4) and affective/motivational (A1, A2, A3, A4). Each scale refers to a specific strategic competence (Table 1). The questionnaire is composed of 46 questions about school and homework. Twenty-one items are grouped in the four cognitive–metacognitive scales as follows: C1 (*n* = 6; e.g., I try to find relationships between what I learn and what I already know), C2 (*n* = 7; e.g., I organize my study based on the time I have available), C3 (*n* = 5; e.g., I build diagrams, graphs or summary tables to summarize what I study) and C4 (*n* = 3; e.g., While the teacher explains, I get distracted). Twenty-five items are grouped in the four affective–motivational scales as follows: A1 (*n* = 6; e.g., I quickly get nervous for a question or problem that I don’t understand immediately), A2 (*n* = 6; e.g., Even if I don’t like the subject, I equally work hard to succeed), A3 (*n* = 8; e.g., When an oral exam goes well, I think I have done well studying hard) and A4 (*n* = 5; e.g., I feel capable of successfully completing my study commitments). The answers were given on a four-point Likert-type scale from 1 (never or almost never) to 4 (always or almost always).

From raw scores for each of the eight subscales, the formula [(sum raw score-mean)/standard deviation × 2 + 5] provided the final profile of students on a nine-point standard Stanine-scale [47]. For the majority of subscales (C1, C2, C3 and A2, A3, A4), the higher the scoring, the more positive the outcome. For the remaining subscales (C4 and A1), conversely, the lower the scoring, the more positive the outcome. Thus, the scores for C4 and A1 subscales were reversed to obtain higher scores corresponding to higher functional skills consistently across scales. According to normative data [47], 1 to 3 are below average skill scores, 4 to 6 points are the average range and 7 to 9 are above average scores. All stanine-transformed scores of each subscale (C1–4 and A1–4) were further transformed in dichotomous variables for data analysis. It was attributed “0” (=no-criticality, i.e., skills above critical level) to all factors which showed a value from 4 to 9 and “1” (=criticality, i.e., skills below critical level) for value from 1 to 3. Summing up the critical values across the four affective/motivational (A1–4) subscales and across the four (C1–4) cognitive/metacognitive subscales [47], each participant could have from 0 (no critical values) to 4 (critical values in all four subscales). Based on this sum of critical values, participants were divided in five (0, 1, 2, 3, 4) categories of criticality in cognitive/metacognitive and affective/motivational skills.

As reported in the validation study [47] performed with 1.182 students, Cronbach’s alpha ranges between 0.59–0.78 across the eight scales, with corrected item–scale correlations being mostly moderate. This Cronbach’s alpha range is deemed acceptable for tools measuring cognitive and affective constructs in learning [48]. A recent Italian study with a larger sample of 3.091 upper secondary school students [49] confirmed a similar Cronbach’s alpha range for the eight scales (0.62–0.78). In the present study, the internal consistency was computed on a larger sample of 1840 Italian and Spanish students (*n* = 1451 and 389, respectively) involved in a wider research project that the present study on youth drop-out is a part of. For the Spanish version of the QSA-R, Cronbach’s alpha was 0.76 for all four cognitive/metacognitive scales (C1–4) and 0.71 for all four affective/motivational (A1–4). Overall, Cronbach’s alpha was 0.76. The values for each scale are reported in Table 1.

### 2.3. Procedure

Data were collected between March and May 2019 by a paper-and-pencil version of the tools. Questionnaires were administered during school time and the compilation of the two questionnaires was performed in a single session (about 20 min) to facilitate the correct matching of tools (and data) for the same student. All students received information for filling out the two questionnaires. Data anonymity was ensured by assigning a unique numerical code to the two questionnaires and an acronym to identify the academic course. Data collection was performed in compliance with school time constraints in order to not overload the students in the self-assessment task and not exceed the maximum time teachers of participant classes agreed on. Students who affirmed that they had never played sports were excluded from the sample, as their data could not contribute to the study of the drop-out phenomenon.

### 2.4. Data Analysis

Descriptive statistics were computed for all variables separately for students who declared drop-out or no drop-out from sports (Table 2).

Frequency analyses were performed to analyze the distribution of sport drop-out rates as a function of: gender, age, nationality, school curriculum, strategic learning skills (affective/motivational and cognitive/metacognitive) and persistence in sport type or change therein. The association of sport drop-out with strategic learning skills (primary focus of the present study) and with persistence in/change of sport (secondary focus) was also analyzed as a function of demographic variables (gender, age and nationality). To this aim, the frequency distribution of drop-out rates was compared among categories of strategic skills deficits (0, 1, 2, 3, 4), and among categories of persistence in/change of sport as a function of gender, age or nationality. After verifying the non-normality of the variables analyzed through the Kolmogorov–Smirnov test (*p* < 0.001), to compute both main and associated effects, bivariate analyses with contingency tables and the chi-square test (*χ*^2^) were conducted. Effect sizes were also computed (phi-coefficient, *φ*, for 2 × 2 contingency tables and Cramer’s *V* for contingency tables other than 2 × 2). Data were processed by SPSS Statistics software (25.0; IBM, Armonk, NY, USA). 

## 3. Results

As reported in Table 2, students dropped-out of sport at an average age of 13.4 ± 1.6 years. A significantly greater dropout of females compared to male students was found, whereas no effect for age emerged. Findings also revealed a significant effect for nationality, with greater drop-out from sports for Spanish as compared to Italian students. However, further analyses distinguishing between sport and non-sport high school curricula in the Italian sample revealed that the cross-country effect depended on the sample composition of school type, which is different in Italy and Spain. The drop-out rate was negligible in Italian students attending sports high schools with enhanced curricular physical education/sport as compared to both Italian students attending high schools with a focus different from sport (such as scientific, applied sciences and linguistic schools) and Spanish ones (Table 2).

Most importantly, an association of drop-out rates with affective–motivational strategic learning skills was found. These skills are associated with drop-out rates independently of gender and age, but interactively with nationality (Table 3). The overall association model showed an increase in the frequency of students who dropped out of sport with the number of critical values in affective–motivational strategic learning skills. As shown in Table 3, this pattern of differences was more pronounced and significant in Italian students, with a progressive increment in drop-out rates from none to all critical values, and less pronounced and non-significant in Spanish students. Cognitive–metacognitive skills were non-discriminant for sport drop-out rates both in Italian (*p* = 0.102) and in Spanish students (*p* = 0.872).

A further analysis also showed that a lack of persistence in the same (individual/team) sport type was predictive of higher drop-out rates in males (Table 2). Highest drop-out rates emerged when the change was not between similar sport types (same-individual or same-team sport), but between individual and team sports. A non-significant reverse trend was observed in females, who more often showed drop-out from sport when they persisted in the same sport or individual/team skill sport type (Table 2).

## 4. Discussion

The main purpose of this study was to evaluate the association between early drop-out from sport and strategic learning skills relevant to both sport and academic career in senior high school students. It was expected that a critical value in and, therefore, lack of strategic learning skills functional to academic performance could be a factor related to the early drop-out from sport and, conversely, a high value of such skills could be related to adherence to sport. The presence or absence of an enhanced sport focus in the senior high school curriculum, as well as behavioral habits in extra-curricular sport, as the persistence of training in a given sport, or switching to another sport—of similar or different type—were also analyzed as potential correlates of different drop-out rates. Moreover, we evaluated the individual and joint role of gender, age and nationality with the strategic learning skills and the persistence in/change of sport.

The results of this study are mostly consistent with the literature and add novel information to the relevance of domain-general strategic skills in relation to the dropout phenomenon in the sport domain. In line with Eime et al. [44] and Molinero et al. [8], we found that young people dropped-out of sport during their teenage age (13.4 ± 1.6 years). Regarding gender, a larger drop-out emerged in females than in males. This is in line with evidence reported by some authors [20,50,51], showing that males are more active than females who have higher sport drop-out rates. Additionally, a female’s tendency to be less active than a male is confirmed as a global gender difference [52]. Further research is warranted to analyze the role of gender stereotypes in sport drop-out with relation to sport self-perceptions of value and match/mismatch of the gender-identity with the gendered-nature of a sport [53].

We did not find comparable studies in literature on sport drop-out between Italian and Spanish students; to our knowledge, this is the first comparative study of adolescent populations from these countries. Our results highlighted a less pronounced drop-out in sports high school vs. other Italian and Spanish high schools. The sports high school is currently a school curriculum that is started up in Italy with no comparable curriculum in Spain. This may be one of the reasons explaining why, among the Italian students of the present sample, which included sports high school students, the sports drop-out is less pronounced than among their Spanish counterparts. Speculatively, the sports high school curriculum may have a favorable impact on sport persistence. Future research is needed to address the hypothesis that the targeted methodological strategies in this school curriculum could help student-athletes pursue their dual career [54].

Beyond the descriptive analysis of the drop-out phenomenon in sport, the most important finding of this study has to do with the association between sport drop-out and domain-general strategic learning skills. Our findings showed an association only for the affective–motivational dimension, but not for the cognitive–metacognitive dimension. This suggests that, in adolescence, an insufficient/critical level of affective skills (e.g., anxiety and emotions control), motivational skills (e.g., perception of competence and causal attribution) and volitional skills (e.g., perseverance) has a direct link to the abandonment of sport. Since sport and academic performance are interrelated [39], quitting sport activity means that students cannot capitalize on the beneficial influence of sport on academic achievement [30,41]. We speculatively suggest that the strategic learning skills may be a linking element within this association. This hypothesis deserves future mediation research.

Although there is a lack of literature on strategic learning skills associated with sport drop-out, we found some agreement with evidence on similar skills and maladaptive sport processes (burnout profiles) that allow us to frame a reference theory. Burnout profiles have been defined as a state of sport/school exhaustion related to chronic stress and have been studied in sport and at school jointly [55]. This burnout process involves a lack/insufficient level of skills that are common to the two areas (sport and school), similar to the strategic learning skills, objects of the present investigation. Thus, our suggestions are based on evidence that highlights the relevance of skills as resilience, similar to the concept of volition addressed in the present study. This evidence comes into play in a phenomenon such as burnout in sport that has commonalities with the drop-out from sport investigated here. A stronger risk of sport drop-out is related to a low level of resilience-related skills [55], high level of anxiety [25] and low perception of competence [24]. The critical level of these competences reported in the literature may represent a risk factor comparable to the critical value of affective–motivational strategic learning skills that emerged in our study. Conversely, athletes with an internal locus of control are more likely to exercise regularly [56], feel that their skill level is appropriate and tend to be more autonomously motivated towards their sport than athletes who drop-out [57].

Although the cross-sectional nature of the present study does not allow us to address causality, we hypothesize that bettering the affective–motivational strategic learning skills may enable us to select the most effective learning objectives and strategies, improving decision making to monitor and evaluate the progresses achieved, engage and persist over time to achieve goals successfully [36]. Being unable to face life transitions—in sport as well as in school and work settings—may foster maladaptive behaviors and negatively impact sport habits [23]. In the sport domain, improving learning processes fosters positive sport experiences; this may, in turn, support long-term sport participation and oppose the drop-out phenomenon. Our findings are in line with the literature showing that self-management of social-cognitive strategies (e.g., goals, plans and acts), similar to skills addressed in this study, may operate as mediators in the association between self-efficacy and physical activity/sports training in order to achieve goals and foster sport persistence [58]. Moreover, personal characteristics, such as personality traits, goal orientation and volitional skills, may contribute in the same way to face critical situations, such as dropping-out or pursuing a career in sport [59,60].

These findings call for studies that investigate the association of strategic skills with sport drop-out longitudinally. Based on the present cross-sectional evidence, it may be hypothesized that developing competences and personal skills common to the sporting and school context may be crucial to achieve excellence in both domains, and to prevent maladaptive consequences such as burnout and drop-out as a behavioral aspect of a broader maladaptive profile. According to Camiré, Trudel and Forneris [61], we believe that a common functional context may be favorable to acquire skills useful both in sport and in school settings. Since school and sport domains are interrelated [55], and strategic learning skills are transferable and general [36], integrating them in learning processes [62] from early schooling age may help capitalize on these skills later in life—such as, but not limited to, sport context—to achieve performance goals.

The outcomes highlighted that the association between strategic learning skills and drop-out rates could be influenced by nationality, but not by gender and age. This nationality effect leads us to suppose that factors other than strategic learning skills may intervene in Spanish students’ sport drop-out processes. This latter aspect deserves an in-depth analysis of the potential intrapersonal, interpersonal, cultural and societal factors that may underlie the observed cross-country differences. Alternatively, the imbalance in sample size between Italian and Spanish students may have caused insufficient power in the case of the Spanish sample, thus not allowing us to draw definitive conclusions of cross-country differences in sport drop-out.

A second aim of the study was the role played by the persistence in/change to another sport in the students’ sport history. A lack of persistence in the same (individual/team) sport type was predictive of higher drop-out rates, but only in males. Sampling different sports until about 12 years (the so-called ‘sampling years’) develops fun and participation [63]. Successively, in adolescence (specialization years), persisting in the same sport seems appropriate for males to pursue personal achievements, maintain individual interest, re-engage and persist in sport [63]. Playing the same sport type probably motivates them to persist in sport activity to excel and win in competition supported by a performance orientation [64]. Following a cascade process, the greater the fun in sports competitions, the greater the increase in fun of sports training and the persistence in sports over time [65]. Females show an opposite tendency, suggesting that a more process-oriented approach to sport and a stronger motivation to learn different skills without a primary performance goal might render females more persistent in sports if they play different sport types. Reasons for the higher drop-out rates of females may include a weak perception of their own competence associated with sport demand and pressure [23]. Indeed, those females who perceive themselves as athletic are more likely to maintain participation in sport [8]. To understand this difference between males and females in sport persistence/abandonment, other determinants or moderators, such as motivational aspects, must be considered in the future.

This study is not without limitations. Although our large sample size (*N* = 614) and stratified sampling ensured representativeness, the homogeneity of the prevalent socio-economic status of the urban districts and the selected schools were located in limits of the generalizability to youths living in different socio-economic contexts, typically influencing their physical activity and sport habits. This similarity allowed us to dampen the influence of potential major socio-economic covariates and to compare cross-country and cross-school system differences and their impact on sports habits. Moreover, a more balanced sample in the two countries could allow for a stronger interpretation in terms of cross-country differences in the association between sport drop-out and strategic skills, excluding power issues that may have undermined the possibility to detect this association in the smaller sample of Spanish students.

This study is cross-sectional and therefore does not allow any causal inference. However, its results pave the way for an intervention study aimed to clarify the causal relationship between strategic learning skills and sports abandonment, sport participation and academic achievement and the role played by gender, nationality and sport type. Finally, this study was conducted only in two cultural settings—Spain and Italy—within their specific schooling contexts. It would be important to implement this study in wider multi-national cultural settings.

## 5. Conclusions

This study suggests that the affective–motivational strategic learning skills relevant to sport and non-sport life domains may play a role in relation to youths’ drop-out. Moreover, the cultural and personal characteristics, and sports habits, may independently and jointly contribute to the phenomenon of drop-out. We believe that these interrelations must be considered to develop targeted strategies for prevention of drop-out, starting as early as schooling. We assume that, similarly to any life skills, strategic learning skills are not learned automatically, but can and should be taught in appropriate contexts, such as school and sport [66], through meaningful experiences [40]. Sport programs tailored to include this aim might be more successful in promoting youth sport participation, optimizing performance and preventing drop-out with positive consequences for talent identification and promotion. Specifically, in adolescents, reflective processes on their strategic learning skills should be encouraged. This may help the student-athlete develop self-determination and self-regulation, in order to be able to monitor and evaluate the learning progression, orient themselves in study and sport and build a successful and long-term continuous developmental path.

## Figures and Tables

**Table 1:** QSA-R: Scales and Dimensions.

Dimensions	Scales		C. α *
Cognitive Metacognitive	C1	Processing strategies for understanding and remembering	0.74
	C2	Self-regulation strategies	0.64
	C3	Graphic strategies to understand, summarize, and memorize	0.71
	C4	Attention control strategies	0.63
Affective Motivational	A1	Emotion management strategies	0.79
	A2	Volition	0.74
	A3	Causal attribution-Locus of control	0.52
	A4	Perception of competence	0.72

Note: * Cronbach’s alpha of 1840 Italian and Spanish students.

**Table 2:** Frequency of students who dropped out/did not dropped out of sport as a function of age, gender, nationality, school curriculum, sport type and strategic learning skills. Absolute frequencies and percentages (*N*/total of each corresponding category) and *χ^2^* test results are reported.

Variables	Drop-Out *N* (%)		No Drop-Out *N* (%)		*χ^2^*	df	*p*	Effect Size
**Age**					4.18	2	0.123	0.083 ^a^
14 years	26 (18.4)		115 (81.6)					
15 years	31 (11.9)		230 (88.1)					
16+ years	37 (17.4)		175 (82.6)					
**Gender**					6.35	1	0.012	−0.102 ^b^
Male	45 (12.3)		321 (87.7)					
Female	49 (19.8)		199 (80.2)					
**Nationality**					13.69	1	<0.001	−0.149 ^b^
Italian	50 (11.7)		376 (88.3)					
Spanish	44 (23.4)		144 (76.6)					
**School curriculum**	1 vs 2				45.29	1	<0.001	−0.326 ^b^
	1 vs 3				47.38	1	<0.001	−0.342 ^b^
1. Italian: sports high schools	3 (1.4)		213 (98.6)					
2. Italian: other high schools *	47 (22.4)		163 (77.6)					
3. Spanish: compulsory secondary school	44 (23.4)		144 (76.6)					
**Type (individual/team) and amount of sports practiced**								
Males					13.87	2	0.001	0.224 ^a^
Only one sport	10 (8.3)		110 (91.7)					
Multiple sports of one type	13 (14.4)		77 (85.6)					
Multiple sports of different type	19 (28.8)		47 (71.2)					
Females					1.59	2	0.450	0.089 ^a^
Only one sport	15 (28.3)		38 (71.7)					
Multiple sports of one type	25 (21.9)		89 (78.1)					
Multiple sports of different type	6 (17.1)		29 (82.9)					
**Strategic Learning Skills**								
Cognitive/Metacognitive								
Scales	No CL **	CL **	No CL	CL				
**C1**	63 (67.0)	31 (33.0)	361 (69.4)	159 (30.6)	0.21	1	0.643	−0.019 ^a^
**C2**	66 (70.2)	28 (29.8)	379 (72.9)	141 (27.1)	0.28	1	0.594	−0.022 ^a^
**C3**	68 (72.3)	26 (27.7)	339 (65.2)	181 (34.8)	1.82	1	0.177	0.054 ^a^
**C4**	38 (40.4)	56 (59.6)	247 (47.5)	273 (52.5)	1.60	1	0.206	−0.051 ^a^
Affective/Motivational								
**A1**	69 (73.4)	25 (26.6)	428 (82.3)	92 (17.7)	4.09	1	0.043	−0.082 ^a^
**A2**	34 (36.2)	60 (63.8)	236 (45.4)	284 (54.6)	2.74	1	0.098	−0.067 ^a^
**A3**	56 (59.6)	38 (40.4)	371 (71.3)	149 (28.7)	5.20	1	0.022	−0.092 ^a^
**A4**	62 (66.0)	32 (34.0)	417 (80.2)	103 (19.8)	9.40	1	0.002	−0.124 ^a^

a = Cramer’s V; b = φ-coefficient; * Scientific, applied sciences, linguistic senior high school; ** No CL: no criticality (below critical level) = “0”; CL: criticality (above critical level) = “1” (dichotomous variables).

**Table 3:** Frequency of students who dropped out of sport as a function of the amount of critical values in affective-motivational strategic skills. Absolute frequencies and percentages (N/total of each corresponding category of critical values) and *χ^2^* test results are reported.

Sample	Amount of Critical Values						*χ^2^*	df	*p*	Effect Size
	0 N (%)	1 N (%)	2 N (%)	3 N (%)	4 N (%)	Total N (%)				
Italian	8 (6.2)	16 (10.7)	12 (14.1)	11 (22.9)	3 (21.4)	50 (11.7)	11.50	4	0.021	0.164 ^a^
Spanish	6 (15.3)	15 (23.1)	16 (28.1)	5 (20.8)	2 (66.7)	44 (23.4)	5.31	4	0.256	0.168 ^a^
Total	14 (8.3)	31 (14.4)	28 (19.7)	16 (22.2)	5 (29.4)	94 (15.3)	13.83	4	0.008	0.150 ^a^

a = Cramer’s V.

## Data Availability

The data presented in this study are available on request from the corresponding author. The data are not publicly available, as they are part of a larger research project not yet completed.
